# Supplementary material for: Left-Right Asymmetry in Predicting Brain Activity From LLMs’ Representations Emerges With Their Formal Linguistic Competence
Source: Neurobiol Lang (Camb). 2026 Sep 4;7:NOL.a.284. doi: 10.1162/NOL.a.284 (PMC13612846; doi:10.1162/NOL.a.284)
Supplement: Supplementary file 1 [file nol-07-284-s001.pdf]

Supplementary material for the paper:

Left-right asymmetry in predicting brain activity from LLMs' representations emerges with their formal linguistic competence

1 Supplementary Figures

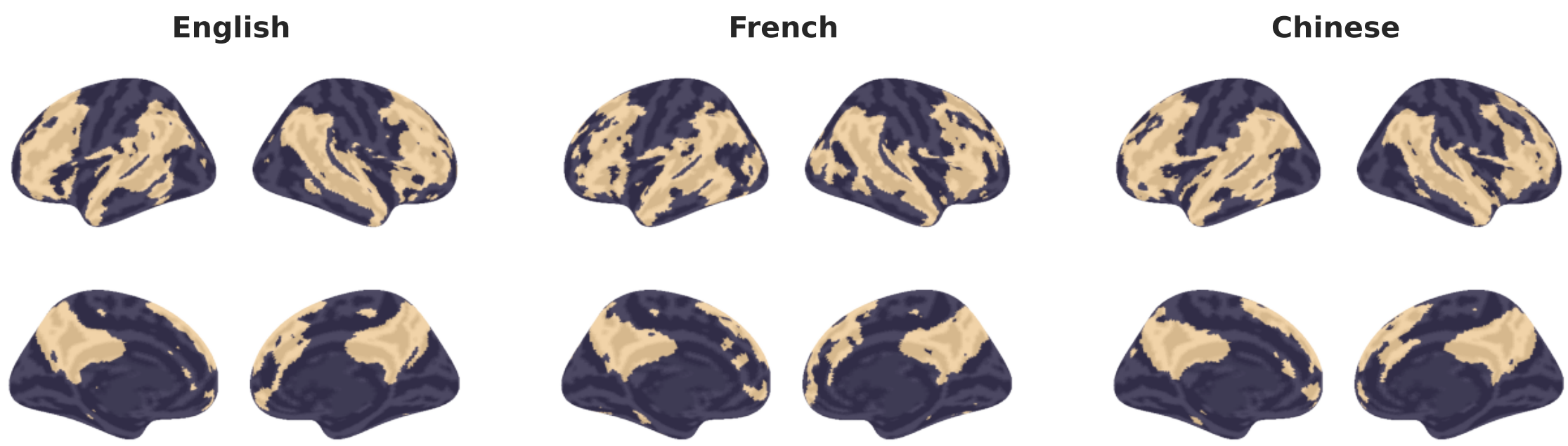

Figure S1: **Cortical surface renderings of the masks used to extract brain predictivity scores used in all main figures of the paper**. The masks encompass the voxels having the 25% strongest inter-subject-correlations within a whole brain mask (the figures here do not display the cerebellum, which also included some voxels with high ISC). Note that these masks, obtained from three independent datasets (different machines, different participants, different languages), are similar, essentially symmetrical and encompass the core temporo-frontal language network.

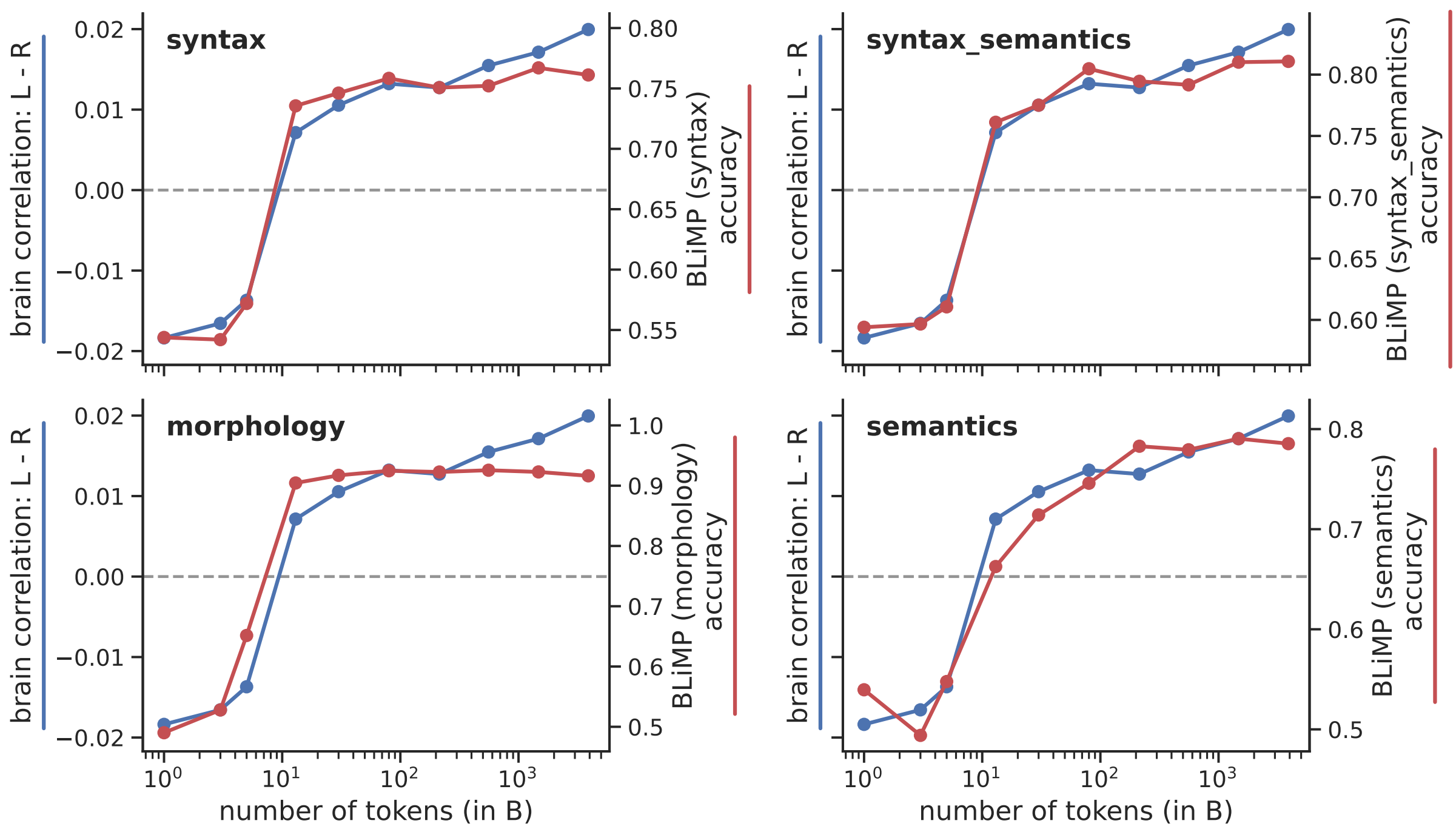

Figure S2: **Results split by BLiMP subtasks**. BLiMP categorizes minimal pairs into four different fields (“syntax”, “syntax_semantic”, “morphology”, “semantic”). For each field, a panel displays the evolution of performance on the corresponding minimal pairs, along with the left-right brain predictivity asymmetry (as in Fig. 1).

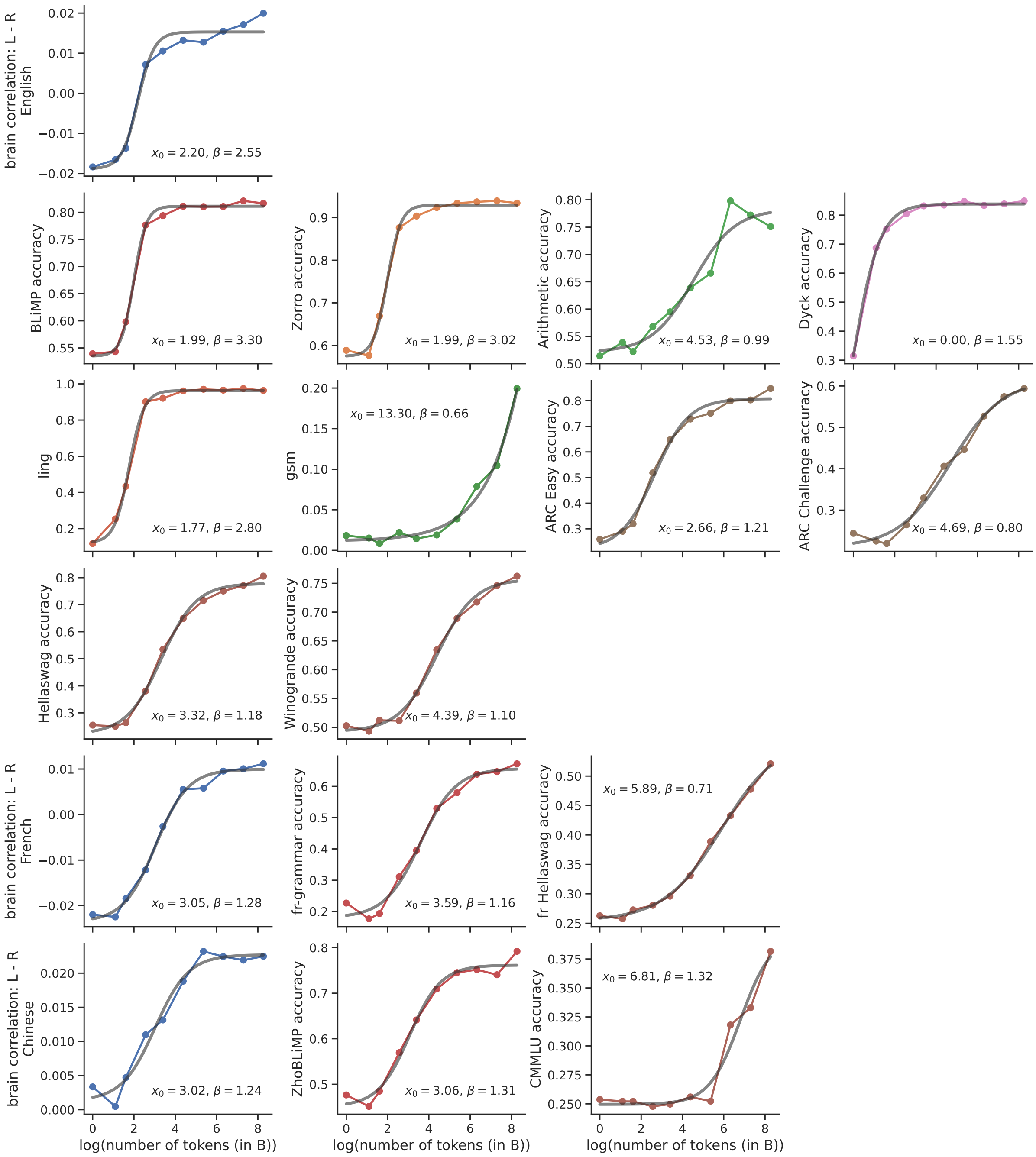

Figure S3: **Sigmoid fits to the evolution of various quantities during the training of the OLMo-2 7B language model**. The colored lines correspond to empirical data, while the gray lines are the fitted sigmoidal curves. The x_0 and β (slope) parameters estimated from these fits were used to construct Fig. 3.

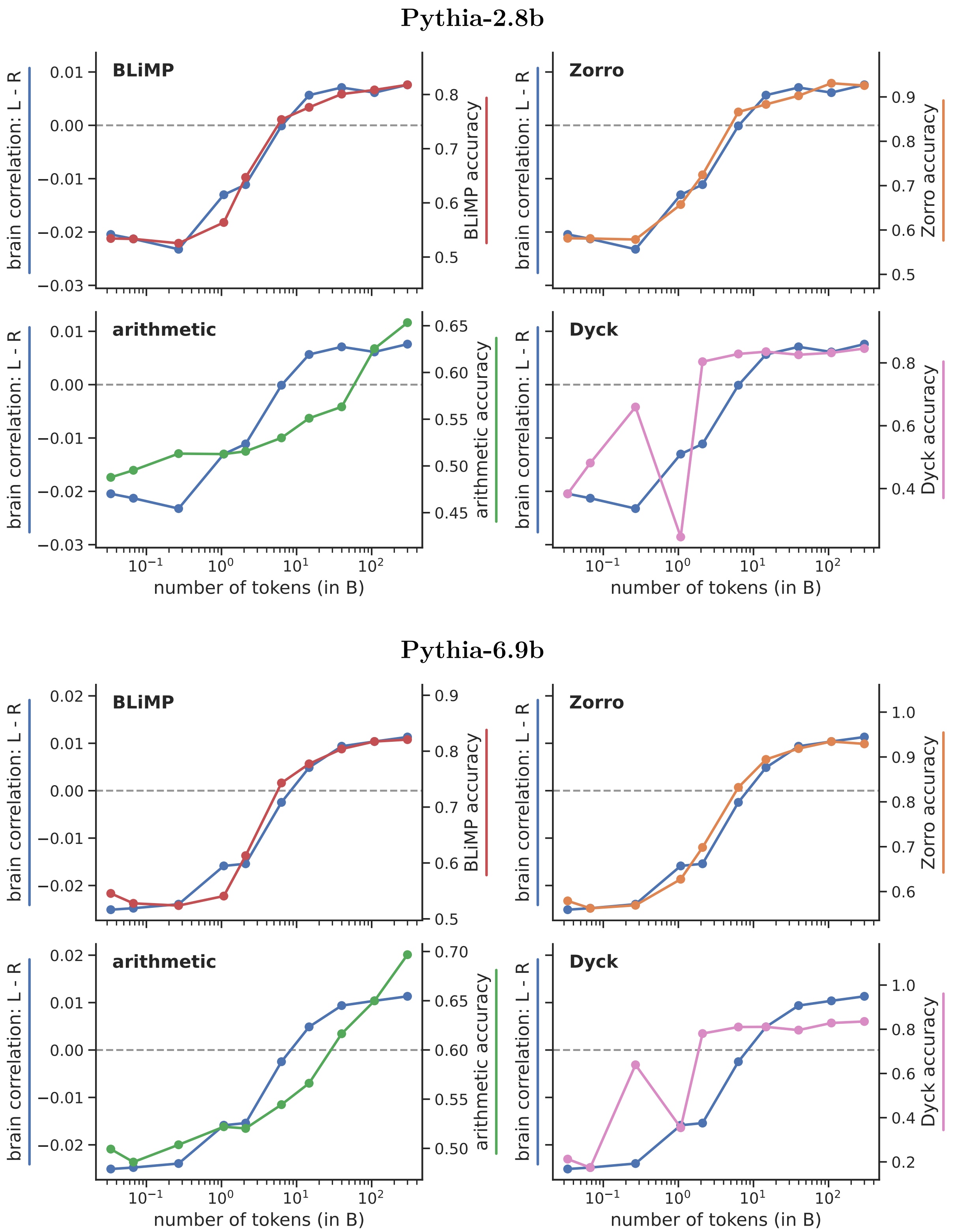

Figure S4: **Generalization to other language models.** Same as Fig. 1 but for the Pythia-2.8b (top panel) and Pythia-6.9b (bottom panel) models. In each case, the trajectory of left-right brain asymmetry aligns well with the evolution of the performance on linguistic minimal-pair benchmarks, but not on the non-linguistic ones.

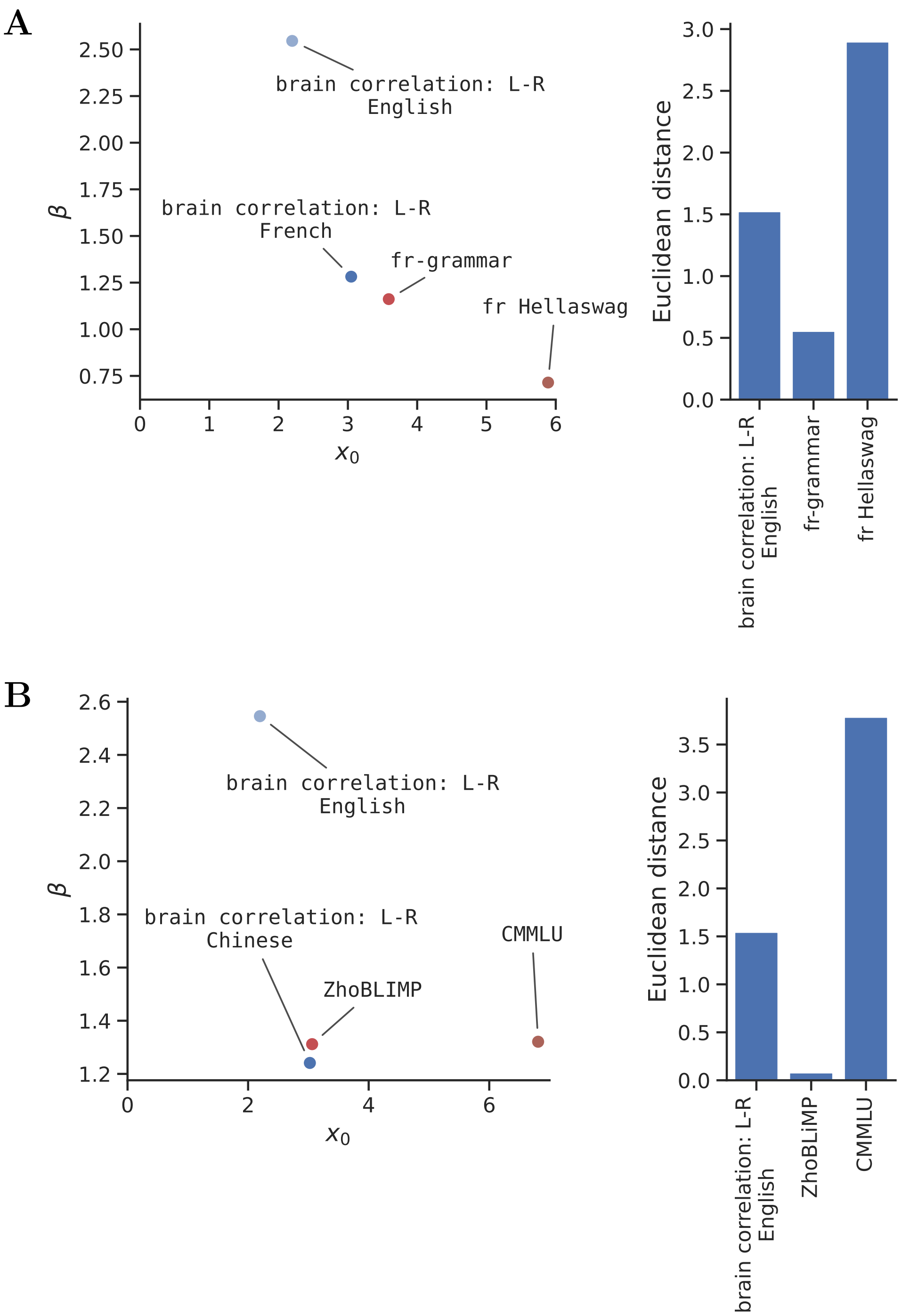

Figure S5: **Quantitative comparison of the evolution of the left-right hemispheric brain correlation in French (A) and Chinese (B) participants and the various performance trajectories.** Similar analysis as in Fig. 3 but for the French and Chinese data. Left-right asymmetry in English participants is also provided as a comparison. The right panel shows the Euclidean distance between the location on the (x_0, β) plane of each point on the left panel and the left-right asymmetry in French or Chinese participants. See Fig. S3, bottom rows, for a full visualization of the sigmoid fits.

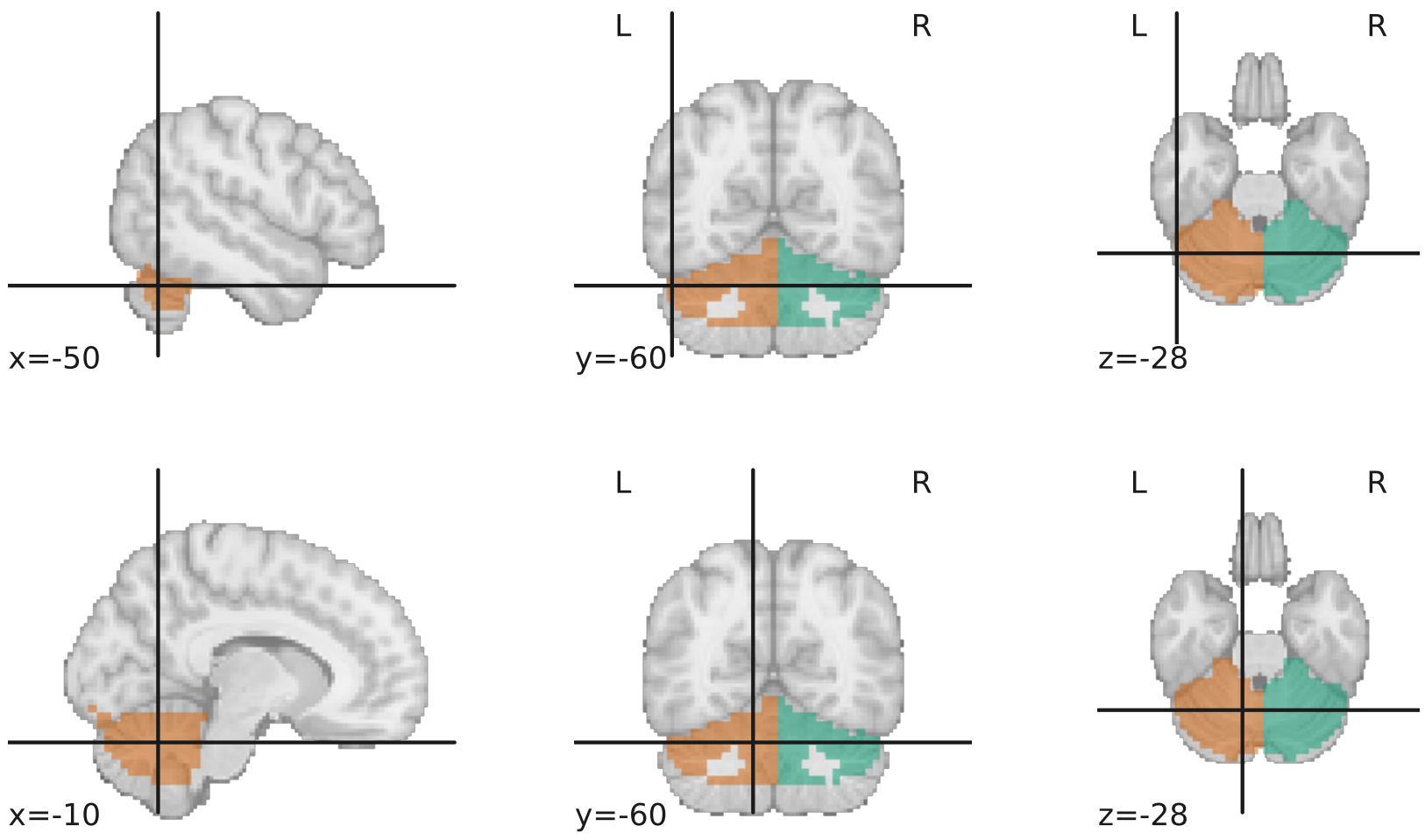

Figure S6: **Left and Right Cerebellum masks.** These masks were obtained from the symmetric BASC atlas scaled at 12 (Bellec et al., 2010).

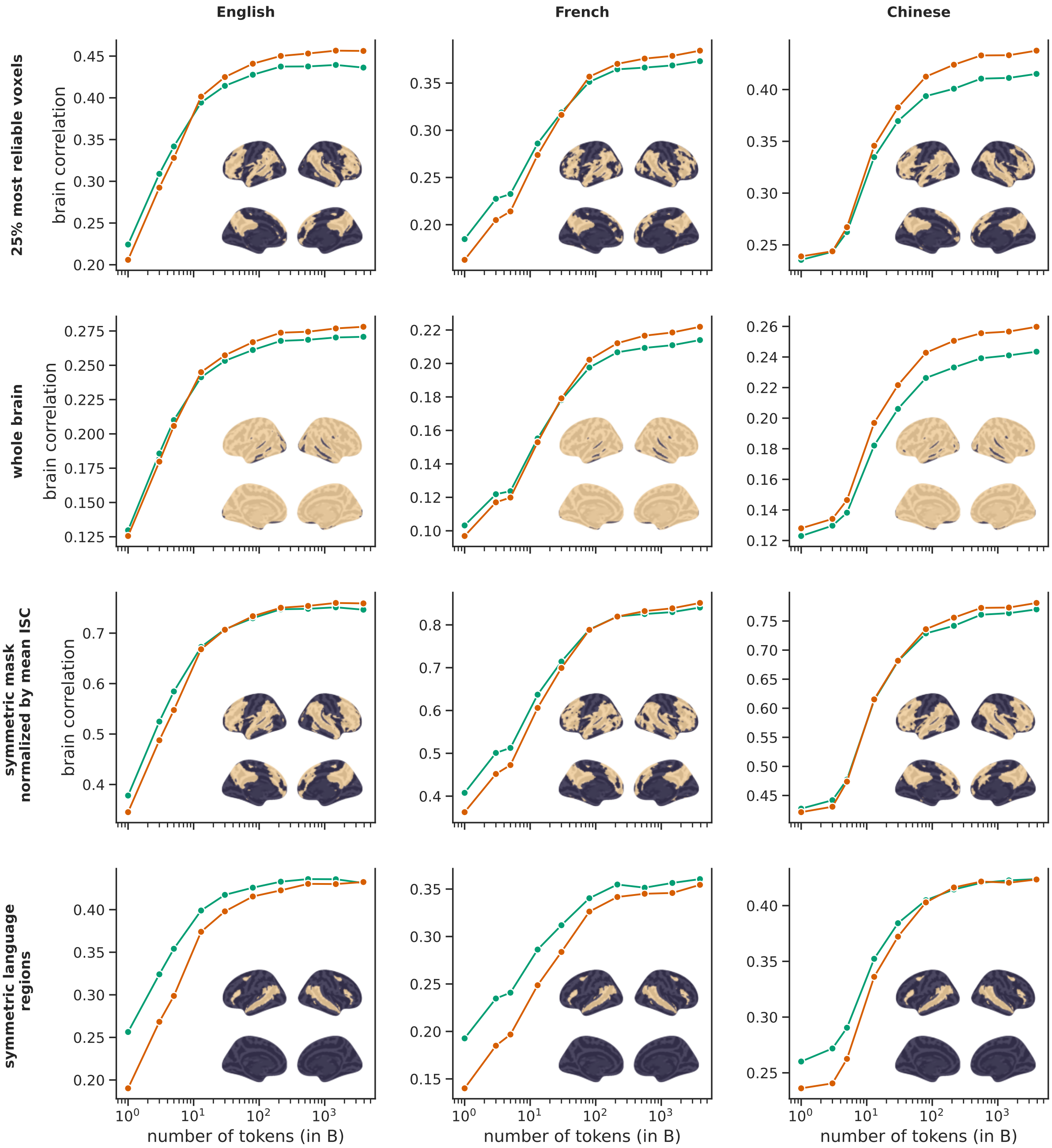

Figure S7: **Hemisphere-specific brain predictivity values (left and right separately), for various masks.** Each column corresponds to a different language (computed from its corresponding text and fMRI average subject). (First row) Main mask used in the study, made of the 25% voxels with the highest inter-subject correlations, also presented in Fig. S1. (Second row) Whole brain. (Third row) Symmetric mask computed by taking the 25% voxels with the greatest inter-subject correlations for each hemisphere separately, then combining them and symmetrizing the mask. The mean brain correlation for each hemisphere is then normalized by the mean ISC in its corresponding hemisphere. (Fourth row) Symmetric mask of language areas, from the parcels provided by Fedorenko lab, which are updates of the original parcels described in (Fedorenko et al., 2010). As for Fig. S1, the masks are presented on the surface of the cortex only, but for the first three cases, there are also voxels in other brain regions, in particular in the cerebellum.

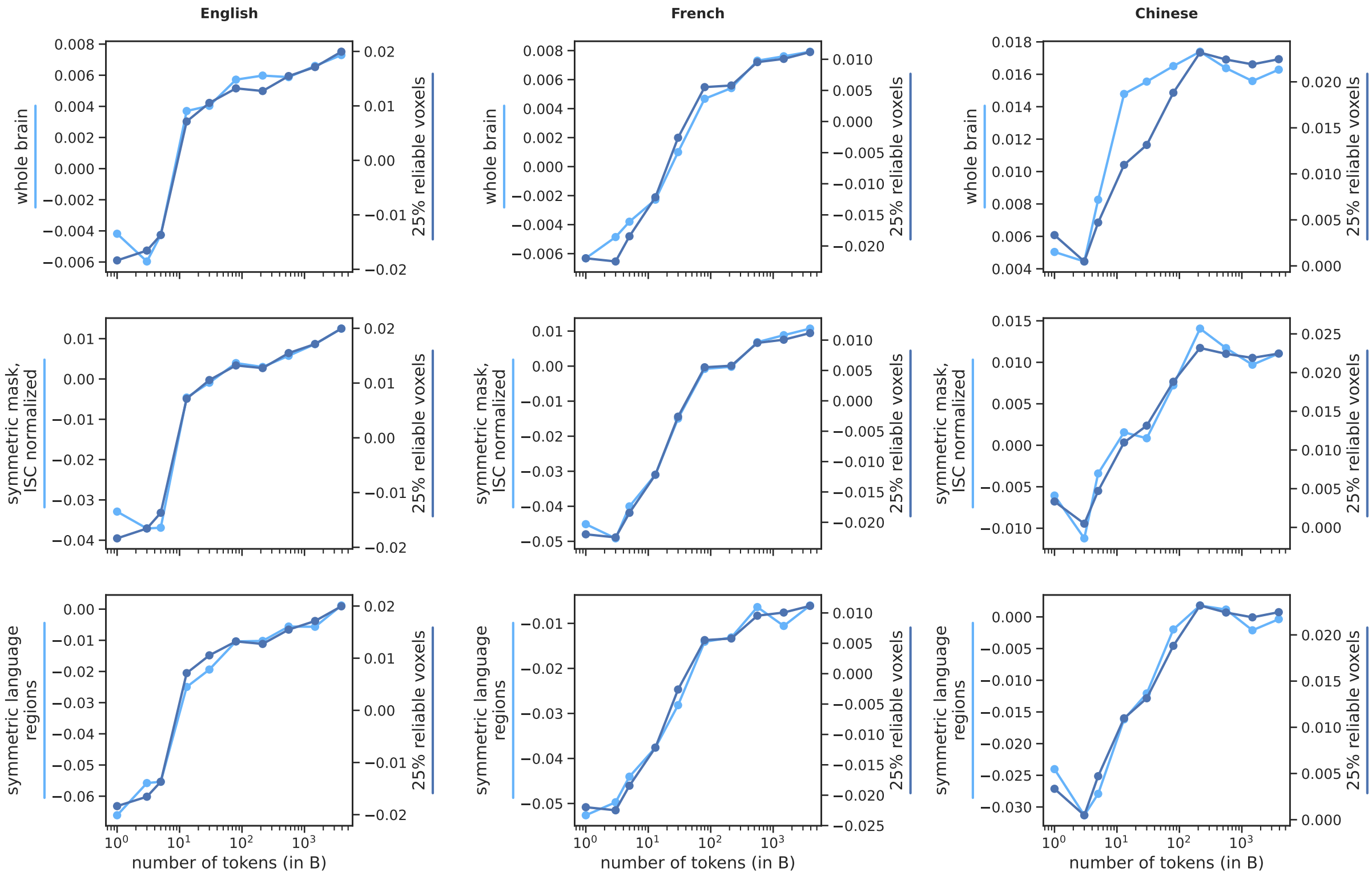

Figure S8: **Robustness to mask choice: comparison of various methods to compute left-right asymmetry**. The lighter blue curves, associated with the left y-axis, are variants of the left-right asymmetry computed from the masks displayed in Row 2 to Row 4 of Fig. S7. The dark blue dotted curves represents the left-right asymmetry used in the paper, computed on the 25% most reliable voxels for each language. Across the methods (whole brain, symmetric mask + ISC normalization, language regions), the left-right asymmetry in the correlation between brain activity and model prediction has a robust shape. The only exception concerns the Chinese dataset for the whole-brain mask: the left-right asymmetry emerges a bit earlier than with the other variants. Focusing on 'unreliable' voxels indeed shows a spike at this location during training, as shown in Fig. S9.

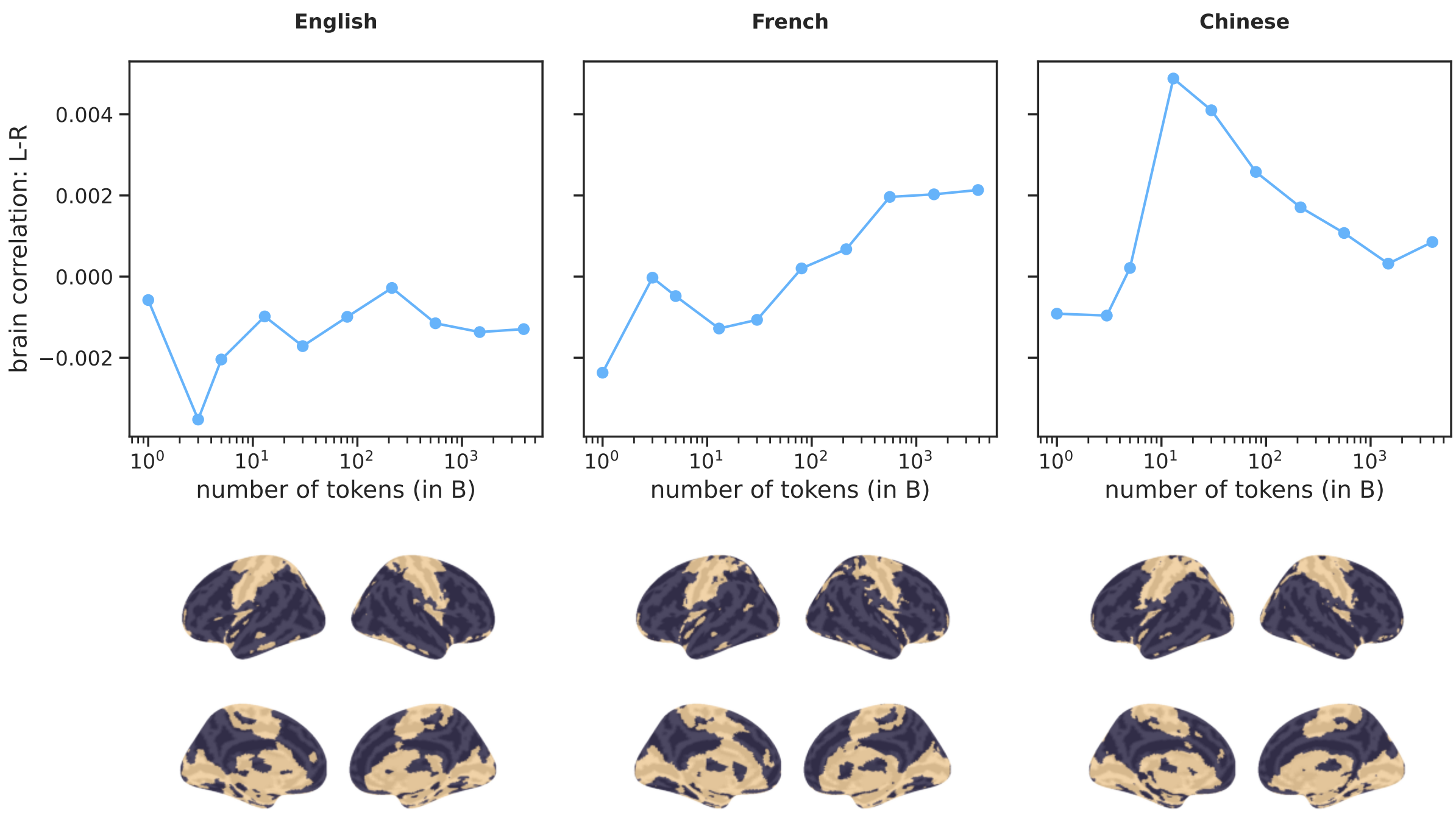

Figure S9: **Left-right asymmetry computed on the 50% least reliable voxels.** The voxels are located in regions such as visual or motor cortex. In the case of Chinese, we observe an unexpected spike at the time during training of the discrepancy between whole brain and all the other methods of computing the left-right asymmetry, as seen in Fig. S8.

2 Examples of texts generated by OLMo-2 7B for different training checkpoints

This section provides examples of texts generated by the OLMo-2 7B model for the 10 different training checkpoints selected for analysis in the present study, from a given prompt ("Are you") and a given random seed. Only the first 500 characters of each text are displayed to save space. Each sample starts with a number (1B, 3B, 5B, etc) indicating the number of tokens (in billions) seen by the corresponding checkpoint.

All generated texts will be provided in the `generated_texts.zip` file available on the GitHub page of the project. Because we noticed some texts contain harmful, toxic, or explicit content, we used a custom Python script running ShieldGemma[1] (Zeng et al., 2024) to automatically filter them out.

```
-------------------------------------------- 1B --------------------------------------------

Are you is you your.  " in what to you, you, or they, in, be.  m at, you have out, He was.

", you are, but, and, " In the " per of a of her, you need your these you, in., and at are a to
no that your about in the same on it, but.  The a, but you need is the the about her it on the
the on the The way with the way you a " "The is an " The other with the the right of the,
but that the way of the 1..   This a  This is the the the first of the their.  This, and that
did the same is no one of some.  "a

-------------------------------------------- 3B --------------------------------------------

Are you is you are trying to the people to believe he has to believe they cannot be able to
believe their behavior.  "What are you here?"" cannot you give you to a young day." He can be
a part of a world, like a boy, who is in the same room at a time to avoid getting your eyes in
the face to take care of themselves that is not so important.  In fact, we must know that if
your son will make your own thoughts, you should try and see if you want to make that decision?
It is not enough to take a walk

-------------------------------------------- 5B --------------------------------------------

Are you would go back for the first day to read, you probably don't know what you don't want at
your own pace.

And it was really interesting, you could be telling, 'Don't give them some big thumbs,'" she
said.

"He's a big stick maker, just like 'Don't know where he's going.'  And the things on it, I
don't remember where you got off so long.  And what it really helped me on was to have the
opportunity to read everything we have, and to have an idea that's what you want to be doing,
it takes the t

-------------------------------------------- 13B --------------------------------------------

Are you using the new method of the day to help your clients, or are you using the old way?

A few years ago when I was doing some consulting, I was telling people I had worked in the
past, and I was telling them I'd been in the industry 12 years, and at that point I'd just kind
of left the company.  But, I don't remember any of the stories or the details, but it seems
like I did get to have the opportunity to go into a variety of things at one point.  I got to
travel for a long period of time, I

-------------------------------------------- 30B --------------------------------------------

Are you using the new Google Mail?  This is a great way to keep the email conversation in the
same place with just a few clicks.
```

[1] Available at https://huggingface.co/google/shieldgemma-2b

For the uninitiated, Google is a search engine, email service, and a lot more. And this new method is a perfect way to make Google Mail better and less intrusive for our everyday life.

Here we'll take you through the steps.

1. Sign up

For our email account, the most efficient way to open Google Mail is through a Google account (see here). Once signed up you will h

-- 80B --

Are you so sure your child won't be happy here, you've got to send them somewhere else. Or at least you have to make it so miserable that they'll leave."

She smiled and put her hands on her hips. "Is that what you think your job is here in the school?"

He scrunched up his face. "No, actually it's about getting that kid to behave. He is completely out of control. He's disruptive on the school bus. He throws things at teachers and students."

She smirked at that one. It was one of the funnier pa

-- 214B --

Are you using the new Google Play Services SDK to develop your apps, or are you using the older GCM API? GCM: We use the older GCM.

Do you have a best practice for apps who will use the Google Play Services SDK? Don't use it. We had some performance issues and we're working with Google to make the SDK better. We won't use it.

What's the performance issue? For some apps on Android devices, they run out of memory and have problems. It's a Google issue, though, not an Ionic issue.

Android is a v

-- 558B --

Are you using the new Google Analytics 4 to track your clients, or are you using the normal Google Analytics? How does it compare?

For the complete overview, you are going to want to download the free report which contains all this information. But you need a link to Google in order to do this. This is where our free 3-in-1 Google tool could come in.

1. In order to download the Google Analytics 4 free report, follow the instructions below: 2. Click on your profile picture in the top right

-- 1477B --

Are you using the new Google Play Music service to play music you've purchased with the Google Music store? Or are you using the legacy, store? It may be time to reconsider. Google Music

If you are using the new Google Play Music, you need to upgrade your Google Music. Google is now blocking accounts with older versions of the Google Music app on Google Play from using that version of the app. Why are they doing this? It's pretty simple: to force you to pay for Google Music.

Previously, Google

-- 3896B --

Are you using the new tools we made available to you, you've still got the old ones too. Use the new ones. They make it easier.

"Your customers are telling you, 'Give us the good stuff. I don't want this anymore.' "

That means you want people talking about what they're watching. They're talking about the things they're watching because it's good and because you've given them the tools.

It seems like the most efficient way to engage with people is through a combination of things. You can have

References

Bellec, P., Rosa-Neto, P., Lyttelton, O. C., Benali, H., and Evans, A. C. (2010). Multi-level bootstrap analysis of stable clusters in resting-state fMRI. *Neuroimage*, 51(3):1126–1139.

Fedorenko, E., Hsieh, P.-J., Nieto-Castañón, A., Whitfield-Gabrieli, S., and Kanwisher, N. (2010). New method for fMRI investigations of language: defining rois functionally in individual subjects. *Journal of neurophysiology*, 104(2):1177–1194.

Zeng, W., Liu, Y., Mullins, R., Peran, L., Fernandez, J., Harkous, H., Narasimhan, K., Proud, D., Kumar, P., Radharapu, B., Sturman, O., and Wahltinez, O. (2024). Shieldgemma: Generative AI content moderation based on Gemma.
